# Commentary: A lymph node mediastinal foreign body reaction mimicking nodal metastasis: a case series

**DOI:** 10.3389/fmed.2023.1259819

**Published:** 2023-09-26

**Authors:** Gianluca Franceschini

**Affiliations:** ^1^Fondazione Policlinico Universitario Agostino Gemelli IRCCS, Rome, Italy; ^2^Department of Woman and Child Health and Public Health, Rome, Italy; ^3^Università Cattolica del Sacro Cuore, Rome, Italy

**Keywords:** Oxidized Cellulose, surgery, haemostatic agent, complications, foreign body reaction, lung, misdiagnosis

## Introduction

I read the article of Lina Zuccatosta and colleagues entitled “*A lymph node mediastinal foreign body reaction mimicking nodal metastasis: a case series*” and published on “Frontiers in Medicine” (1).

The Authors report a series of eight patients with enlarged on CT and PET-FDG positive lymphadenopathies due to foreign body inflammatory reaction to Oxidized Cellulose (OC) after lung surgery (1).

I would like to take inspiration from this article in order to urge the scientific community about other possible serious problems associated with the use of OC as haemostatic biomaterial in surgery.

OC is derived from plant-derived cellulose and is treated with oxidizing agents to create a product that helps control bleeding in surgery (2).

However, even if OC is widely used and generally considered safe, there are some potential problems associated with its use that should be carefully considered.

## Issues related with the use of OC

- Allergic reaction: although rarely, some patients may be allergic to OC and experience localized skin reactions or more severe systemic allergic reactions; A 10% rate of allergic skin reactions, presenting with rash, itching, redness, irritation and hives, was observed after breast conserving surgery (BCS) (3, 4).
- Seroma: OC may contribute to the formation of seroma, which is a pocket of fluid that accumulate at the surgical site due to a redundant OC digestion; significant postoperative seroma, resolved within weeks with repeated percutaneous aspirations, was reported in 45% of patients after BCS (2–4).
- Surgical site infection: OC may act as a foreign body and create a breeding environment for bacteria with an increased risk of infections; some cases of abscess formation that required surgical drainage were reported in literature (3, 5).
- Delayed wound healing: the presence of OC within the surgical site may interfere with the migration of cells, collagen deposition, and angiogenesis and potentially delay wound closure (5, 6).
- Foreign body reaction: the body's immune system may perceive OC as a foreign substance, leading to an inflammatory response with fibrogenic reaction; this reaction may cause the formation of granulomas or fibrous capsules and hinder the healing process; various foreign body reactions due to not optimal bioabsorption of OC, that needed surgical removal, were reported in different types of surgery (5, 7–9); Zuccatosta et al. reported the development of lymph node foreign body reaction due to the use of OC as haemostatic agent in eight patients who underwent surgery for lung cancer.
- Adhesions formation: although OC is designed to be absorbed over time, in some cases, it may not fully degrade, leading to the formation of adhesion between tissues and organs; when OC comes into contact with nearby tissues, it may cause them to adhere together during the healing process; these adhesions may cause complications such as nerve compression, pain and interference with normal tissue function (5, 8, 9).
- Ischemic complication: overuse or improper placement of OC at surgical site may also lead to excessive clot formation and limited absorption that can potentially impede normal tissue perfusion and cause ischemic complications; in this regard, some Authors observed a full-thickness necrosis involving the epidermis up to the periosteal layer when OC was applied above the periosteum to control bleeding and prevent haematoma formation after a scalp laceration (10).
- Misdiagnosis during follow-up: sometimes, the OC-induced fibrogenic reaction may create a three-dimensional fibrotic structure with a peculiar imaging that leads to diagnostic misinterpretation during postoperative follow-up (11, 12).

## Discussion

Every surgeon must be aware of the aforementioned problems and adopt some appropriate precautions to mitigate them, such as (13):

- Careful patient selection: the use of OC should not be considered in patients with some specific pathologies (e.g., uncontrolled diabetes mellitus and immune disease) that have an increased risk of postoperative infections.
- Proper technique of OC application: OC should not be used directly under the surgical suture to avoid the risk of its extrusion; the amount of OC to be used should be calibrated to prevent overdose; OC should adequately fill the surgical cavity but it should not be oversized to avoid excessive fibrosis and foreign body reactions.
- Strict compliance with rules of asepsis: the OC should be handled with great caution and antibiotic therapy should be prescribed in the postoperative period to prevent infections.
- Close postoperative monitoring: a careful clinical evaluation of the patient is necessary as the prompt management of postoperative complications, even with possible removal of the OC from the surgical site, should begin as soon as possible in order to minimize problems and optimize results.
- Detailed surgical report: the surgeon should always describe the use of the OC in the surgical report so that radiologists can correctly interpret the imaging and avoid misdiagnoses during follow-up.

In conclusion, while research and technological advances try to overcome the issues associated with OC, skilled surgeons should carefully evaluate the risks and benefits of this biomaterial on a case-by-case basis in clinical practice and, if necessary, also consider alternative haemostatic agents or techniques (gelatin-based agents, chitosan-based agents, kaolin-based agents, collagen-based products, topical thrombin, fibrin sealants, synthetic haemostatic agents and electrical haemostatic devices) (14).

## Author contributions

GF: Conceptualization, Writing—original draft, Writing—review and editing.
